# Quality of reporting and adherence to the ARRIVE guidelines 2.0 for preclinical degradable metal research in animal models of bone defect and fracture: a systematic review

**DOI:** 10.1093/rb/rbac076

**Published:** 2022-10-03

**Authors:** Fengxing Ding, Kaiyan Hu, Xia Liu, Chen Liu, Jinwei Yang, Xinli Shi, Bin Liu, Mei Wu, Zhe Wang, Liyuan Feng, Jiazhen Zhang, Bin Ma

**Affiliations:** School of Basic Medical Sciences, Evidence-Based Medicine Center, Lanzhou University, No 199, Donggang West Road, Chengguan District, Lanzhou 730000, P. R. China; School of Basic Medical Sciences, Evidence-Based Medicine Center, Lanzhou University, No 199, Donggang West Road, Chengguan District, Lanzhou 730000, P. R. China; Department of Pharmacology, School of Basic Medical Science, Lanzhou University, Lanzhou 730000, P. R China; School of Basic Medical Sciences, Evidence-Based Medicine Center, Lanzhou University, No 199, Donggang West Road, Chengguan District, Lanzhou 730000, P. R. China; School of Basic Medical Sciences, Evidence-Based Medicine Center, Lanzhou University, No 199, Donggang West Road, Chengguan District, Lanzhou 730000, P. R. China; Reproductive Medicine Center, Gansu Provincial Maternity and Child-Care Hospital, Lanzhou 730000, P. R. China; Center for Medical Device Evaluation, National Medical Products Administration, Beijing 100081, P.R China; Center for Medical Device Evaluation, National Medical Products Administration, Beijing 100081, P.R China; School of Basic Medical Sciences, Evidence-Based Medicine Center, Lanzhou University, No 199, Donggang West Road, Chengguan District, Lanzhou 730000, P. R. China; School of Basic Medical Sciences, Evidence-Based Medicine Center, Lanzhou University, No 199, Donggang West Road, Chengguan District, Lanzhou 730000, P. R. China; School of Basic Medical Sciences, Evidence-Based Medicine Center, Lanzhou University, No 199, Donggang West Road, Chengguan District, Lanzhou 730000, P. R. China; Center for Medical Device Evaluation, National Medical Products Administration, Beijing 100081, P.R China; School of Basic Medical Sciences, Evidence-Based Medicine Center, Lanzhou University, No 199, Donggang West Road, Chengguan District, Lanzhou 730000, P. R. China; Key Laboratory of Evidence-Based Medicine and Knowledge Translation of Gansu Province, Lanzhou 730000, P. R. China

**Keywords:** ARRIVE 2.0, animal research, degradable metal, bone defect, fracture, systematic review

## Abstract

*In vivo* testing is crucial for the evaluation of orthopedic implant efficacy and safety. However, the translation and reproducibility of preclinical animal experiments are not always satisfactory, and reporting quality is among the essential factors that ensure appropriate delivery of information. In this study, we assessed the reporting quality of *in vivo* investigations that examined the use of degradable metal materials in fracture or bone defect repair. We employed scientific databases, such as PubMed, EMBASE, Web of Science, Cochrane Library, CNKI, WanFang, VIP and Sinomed to screen for *in vivo* investigations on fracture or bone defect repair using degradable metal materials, and extracted both epidemiological and main characteristics of eligible studies, and assessed their reporting quality using the ARRIVE guidelines 2.0. Overall, 263 publications were selected, including 275 animal experiments. The overall coincidence rate of Essential 10 (22 sub-items) and Recommended Set (16 sub-items) were 42.0% and 41.5%, respectively. Based on our analysis, the reporting quality of the published *in vivo* investigations examining fracture/bone defect repair with degradable metal materials was low, and there was a lack of transparent, accurate and comprehensive reporting on key elements of the experimental design and other elements that are meant to avoid bias.

## Graphical Abstract

**Figure:**
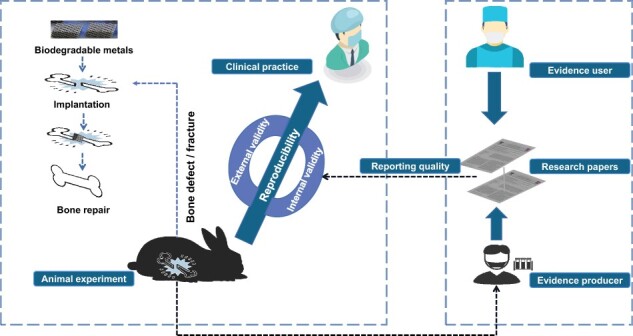

## Introduction

Animal experimentation is an essential bridge that connects basic and clinical research. It is also important for the evaluation of safety and performance of new orthopedic implants [1]. In recent years, the number of bioscience journals increased significantly [2]. But, of particular concern is that numerous animal studies lack appropriate reporting quality [3, 4]. For instance, a study assessing the reporting quality of animal experiments examining urethroplasty revealed insufficient description of key elements of experimental design, such as, how the sample size was calculated (0/6, 0%) and how the experimental animals were allocated to groups (0/6, 0%) [5]. This can seriously affect the validity, reliability and usefulness of research results, and can greatly increase the biological risks in translation of these results into clinical practice and (or) evidence-based guidelines [5]. Similar conclusions were reported in other disciplines, including animal studies in critical care [6], otorhinolaryngology [7], rheumatology [3], sports medicine [8] and so on.

Research papers are not only an important bridge between evidence producers and evidence users, but also the main medium for learning and acquiring knowledge. The characteristics of experimental animals (such as, species, strain, sex, heredity and so on), randomization, blinding, sample-size calculation, statistical method and so on are critical elements of experimental design [9], and it is highly critical for the reproducibility of an animal study to report the above elements both transparently and accurately. Studies with accurate and transparent reporting aid in the complete understanding of the overall process of experimental design and implementation, and it can aid in the scientific and effective review of study methodological rigor, while evaluating the reliability of findings, which is conducive to repeating the previous research or advancing research based on prior investigations [10]. On the contrary, poor reporting quality can not only prevent the successful translation of experimental data to clinical practice, but also diminish the welfare of experimental animals and cause abuse and waste [3].

Medical research report guidelines play a pivotal role in instructing researchers and publishers to report and publish the study design, implementation process and all medical research findings clearly and accurately [11–13]. Up till now, a total of 486 reporting guidelines were formulated and promulgated for different types of medical research [14]. Moreover, increasing number of studies revealed that [15–18], owing to the continuous introduction of medical research guidelines in the journal ‘guide for authors’, the transparency of medical-related scientific research paper reports is being promoted to a great extent. Therefore, NC3Rs, the largest animal experiment funding institution in the UK, issued the Animal Research: Reporting of *In Vivo* Experiments guidelines (hereinafter referred to as ARRIVE guidelines 1.0) in 2010. The guidelines include a checklist of animal experiment information, which facilitate the complete review of animal experiments, along with the evaluation of rigor and reproducibility of study methods. These guidelines were last updated, expanded and reorganized into ARRIVE 2.0 in 2019 [19].

Bone defect and fracture are two orthopedic diseases with elevated incidence and disease burden [20, 21]. In the USA alone, ∼ 8 million people experience fractures every year [22]. Moreover, 5–10% of fractures fail to heal, and develop into nonunion [22]. The average cost of treating nonunion is estimated to exceed $10 000 [23]. In China, nearly 10 million patients each year have bone defects caused by illness, accidents and sports injuries [24]. And in the USA and Europe, more than half a million patients annually receive bone defect repairs with a cost estimated to be greater than US$3 billion [21]. Although bone has a certain ability for regeneration and self-repair, fractures or bone defects, caused by severe trauma, tumor resection, cancer or congenital diseases, can only be repaired via bone transplantation [25]. Therefore, in recent years, the demand for bone graft substitutes is continuing to rise. At present, multiple different materials (non-degradable metal [26], degradable polymer [27], calcium phosphate ceramics [28], etc.) are used to repair fractures or bone defects. Compared to other materials, degradable metal materials garnered much attention in the treatment of fractures and bone defects. This is due to their excellent mechanical property, biocompatibility and degradability [29, 30]. Extensive research was carried out on the repair of fractures or bone defects with degradable metal materials [31, 32]. Previously, we have made a comprehensive analysis of the published animal studies of biodegradable metals repairing bone defects or bone fractures with traditional biomaterials (including non-biodegradable metals, biodegradable polymers, bioceramics and autologous or allogeneic bone grafts) [25, 33]. Evaluating the risk of bias of the included studies is an important step in carrying out a systematic review, and it is also an important means to comprehensively understand and evaluate the reliability and internal validity of experimental results [34]. However, in the process of carrying out our previous two systematic reviews, it was found that the published experiments in the field of degradable metal materials repairing fractures or bone defects did not report sufficient and transparent information on randomization, blind method, sample size calculation and other important factors affecting the internal validity, which may also be one of the main factors that ultimately led to the lack of reproducibility and the obstruction of translation of animal experiments in this field [25, 33].

In the present study, we employed items in the ARRIVE guidelines 2.0 to evaluate the reporting quality of preclinical animal experimentation involving degradable metal materials in treating fracture/bone defects for the first time. This dissertation aims to present the current status of reporting quality in this field, screen and summarize the deficiencies in animal experimentation reports, and promote the reporting quality, reproducibility and translation of animal experiments in this field. Our findings will simultaneously enable researchers, editors, reviewers and other relevant journal staff to gain a better understanding of the report overview of various items in the current guidelines, and encourage relevant journals to introduce ARRIVE guidelines 2.0 into the ‘guide for authors’ to enhance the reporting quality of animal experimentations.

## Materials and methods

### Inclusion and exclusion criteria

#### Inclusion criteria

(i) Population: included studies were animal studies of bone fracture or bone defect; (ii) Interventions: fracture or bone defect repair with degradable metals and their alloys or modified degradable metals and their alloys (composites, coatings and surface modification) [25, 33]; (iii) Controlled studies, without any restriction on randomization.

#### Exclusion criteria

Reviews, conferences, comments, abstracts, non-full-text and non-English/Chinese literature were eliminated from analysis.

### Search strategy

We screened scientific databases like PubMed, EMBASE, Cochrane Library, Web of Science, China National Knowledge Infrastructure or CNKI, Wanfang Data Knowledge Service Platform, Chinese Scientific Journal Database or VIP and China Biomedical Literature Database or CBM from their inception date till October 2020. The retrieval method was the combination of free words and medical subject words (MeSH). The retrieval strategy was ‘(animal studies) AND (degradable metal) AND (bone fracture OR bone defect)’. Table 1 summarizes the search strategy of PubMed [25, 33], and all search strategies using the English and Chinese are provided in Supplementary data 1.

**Table 1.** The PubMed search strategy

Search subject	Key words	Result
#1 Type of study	‘Fractures, Bone’[Mesh]OR fracture*[Title/Abstract] OR Bone defect*[Title/Abstract] OR ‘Fracture Healing’[Mesh] OR ‘Fracture Healing’[Title/Abstract] OR ‘fracture fixation’[Mesh] OR (‘fracture’[Title/Abstract] AND ‘fixation’[Title/Abstract]) OR ‘fracture fixation’[Title/Abstract] OR Bone repair* [Title/Abstract] OR bone heal[Title/Abstract] OR bone healed [Title/Abstract] OR bone heals[Title/Abstract] OR bone healing [Title/Abstract] OR Bone fill*[Title/Abstract] OR ‘Bone Screws’[Mesh] OR ‘Bone Screws’[Title/Abstract]OR ‘Bone Plates’[Mesh] OR ‘Bone Plates’[Title/Abstract] OR ‘Bone Nails’[Mesh] OR ‘Bone Nails’[Title/Abstract] OR intramedullary nail*[Title/Abstract] OR ‘pins’[Title/Abstract] OR ‘Bone Regeneration’[Mesh] OR ‘Bone Regeneration’[tiab] OR ‘Osteoconduction’[tiab] OR ‘bone tissue regeneration’[tiab]	412 099
#2 Object of study	Search filter for animal studies [35]	7 404 572
#3 Intervention	(biodegradable metal OR degradable metal OR biodegradable alloy OR degradable alloy OR absorbable metal) OR ((biodegradable implants OR biodegradable fixation OR absorbable implants OR bioabsorbable implants OR biodegrading implants) AND (metal OR alloy OR magnesium OR Mg OR zinc OR Zn OR Iron OR Fe))	770 893
#4 combination of all key words	#1 AND #2 AND #3 (from 1954 to 2021)	3085

### Literature screening

Two highly trained researchers (F.D. and K.H.) independently employed Endnote X9 to screen and cross-check relevant papers. Disputes were resolved via discussion with a third party (B.M.). In terms of the primary screening, the titles and abstracts were reviewed, according to the pre-set inclusion/exclusion criteria, and subsequently, the full text was reviewed upon exclusion of irrelevant literature to determine the validity of its inclusion. Please refer to the Supplementary document for the full-text screening documents (Supplementary data 2).

### Data extraction

#### Data extraction checklist and method

Two highly trained researchers (F.D. and K.H.) extracted data, according to the pre-set full-text data extraction checklist. This included: (i) epidemiological characteristics of included studies: published journal name, 2020 journal impact factor, year of publication, country of first author and funding source; (ii) major characteristics of included studies: experimental animal disease model, species and strains of experimental animals, sample size, median and interquartile range (IQR) of experimental follow-up time, measuring method and time point of degradation and gas formation outcome indicators, types of degradable metal material and types of control groups. The Supplementary documents (Supplementary data 3–5) provide additional information on our data extraction procedure.

#### Standardization and transformation for data processing

Since the follow-up time (day, week, month and so on) measuring units may be inconsistent between the included studies, we regarded ‘day’ as the statistical measuring unit for the convenience of statistics.

Since the definitions of the control group may be inconsistent between the included studies, we made a unified definition, in accordance with previous studies [25, 33], to better facilitate statistics and present our results. Our results were mainly divided into positive control and other types of controls (Supplementary data 6). The control groups of each study were classified upon reviewing the full text.

Since some studies included two or more animal experiments that met our inclusion criteria, we separately extracted the ‘major characteristics of the included studies’.

### Reporting quality assessment

#### Arrive guidelines 2.0

ARRIVE 2.0 [19] is primarily used to improve the reporting quality of animal experimentation, and it involves 21 items and 38 sub-items. Among them, Essential 10 includes 10 items and 22 sub-items, and these indicate the basic items that must be reported in animal studies (Supplementary data 7). This includes 10 aspects of ‘study design, sample size, inclusion and exclusion criteria, randomization, blinding, measurement result, statistical methods, experimental animals, experimental procedures, and results’. This extensive evaluation enables reviewers and readers to accurately assess the reliability of animal experimentation results and conclusions. The Recommended Set includes 11 items and 16 sub-items, and the items are recommended for reporting in animal experimentation (Supplementary data 7). This includes 11 aspects of ‘summary, background, objectives, ethical statement, housing and husbandry, animal care and monitoring, interpretation/scientific implications, generalisability/translation, protocol registration, data access, and declaration of interests’. Upon the consistent reporting of Essential 10 in the manuscript, items from the Recommendation Set can be included in the journal requirements over time until all 21 items are routinely reported in all manuscripts [10].

#### Methods of assessing reporting quality

Prior to our evaluation, researchers were systematically trained, and the evaluation criteria were fully analyzed and discussed. Additionally, a pre-experiment was conducted to ensure that both parties (F.D. and K.H.) agree on the standards related to the understanding and interpretation of each item (or sub-item). The aforementioned trained researchers independently assessed the reporting quality of 50 included papers using the ARRIVE guidelines 2.0, and they entered the results into an online electronic database (Tencent electronic documents). Following each evaluation of 10 studies, the data were discussed until both parties reached an agreement.

Two researchers (F.D. and K.H.) employed 21 items and 38 sub-items of the ARRIVE guidelines 2.0 to evaluate the eligible literature, and conducted judgments of ‘Yes’, ‘Partly’, ‘No’ and ‘Unclear’, based on the literature content. ‘Yes’ represented that the evaluated literature reported all information related to the corresponding item or sub-item in detail. ‘Partly’ meant that the evaluated literature only partially reported the information related to the corresponding item or sub-item. ‘No’ represented that the evaluated literature did not include any information related to a corresponding item or sub-item. Finally, ‘Unclear’ meant that the reporting quality could not be judged, according to unknown information. Any disputes were resolved via discussion with a third researcher (B.M.).

#### Standardization and transformation methods for inapplicable items

In terms of certain included studies, the following items in the ARRIVE guidelines 2.0 were not applicable for the evaluation of reporting quality:

6b. For hypothesis-testing studies, specify the primary outcome measure: This item applies to hypothesis-testing studies. Upon a complete review of the full text and research purpose of all research included in this study, we concluded that most research experimental types did not belong to the hypothesis-testing category, but belonged to the exploratory research category.

7 Statistical methods: In cases where the research results were qualitative in nature or no statistical analysis was required, this item was evaluated as ‘not applicable’.

10b. If applicable, the effect size was reported, with a confidence interval: In cases where the research results were qualitative data or no statistical analysis was required, this item was evaluated as ‘not applicable’.

Our evaluations based on the aforementioned items are provided in detail in Supplementary data 9. The studies that were not applicable to the above items were not included when we analyze the reporting quality of the above items.

In addition, since not all items were applicable to the evaluation of all included studies, we employed the weighted average formula to calculate the overall coincidence rate ($P_{Y}$ or PY), non-coincidence rate ($P_{N}$ or PN), partial coincidence rate ($P_{P}$ or PP) and unclear rate ($P_{U}$ or PU). Taking the overall coincidence rate ($P_{Y}$) of the ARRIVE 10 Essential (22 sub-items) as an example, the formula we used is as follows:

$$\sum_{i=1}^{22}\frac{m_{i}}{M}\times p_{Y_{i}}$$

$m_{i}$: refers to the number of literature included in the evaluation of each item, $p_{Y_{i}}$ defines the coincidence rate of each item (non-coincidence rate ($p_{N_{i}}$):, partial coincidence rate ($p_{P_{i}}$), rate of unclear ($p_{U_{i}}$), M: sum of the number of evaluated literature per item. Taking the ARRIVE 10 Essential (22 sub-items) as an example, the formula of M is as follows:

$$\sum_{i=1}^{22}m_{i}$$

Finally, it should be noted that even if the selected articles included several animal experiments that met our inclusion criteria, we did not evaluate them separately, but as a whole.

### Statistical analysis

Data from all item evaluations were statistically analyzed in Excel 2019. The enumeration data are expressed as cases and percentages (%), and the measurement data are expressed as mean ± standard deviation (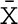
±S).

## Results

### Literature search results

Overall, 7042 articles were extracted from four English and four Chinese databases. Among them, 6797 were in English, and retrieved from PubMed (*n* = 3085), Web of Science (*n* = 2107), EMBASE (*n* = 1567), Cochrane Library (*n* = 38), whereas, 245 were in Chinese, and retrieved from CNKI (*n* = 107), Wanfang (*n* = 113), VIP (*n* = 6) and CBM (*n* = 19). Upon exclusion of duplicate publications (*n* = 1045), 5997 articles underwent preliminary screening. Following the exclusion of inconsistent research types, research objectives, intervention measures and non-Chinese/English literature, a total of 413 valid publications were entered into the full-text screening process.

**Figure 1.**
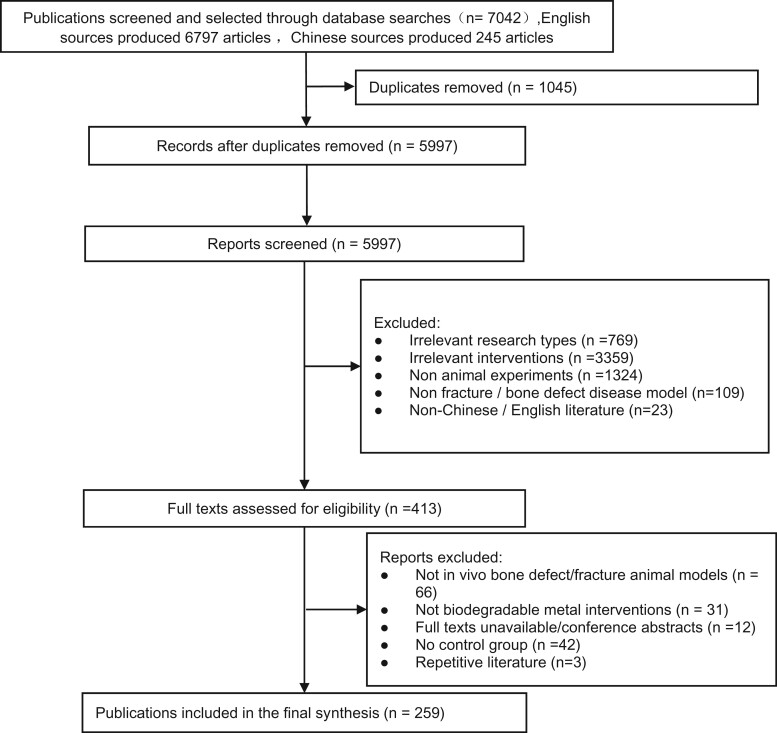
A flow chart of the study screening and selection process.* *It is worth noting that during the research process, two searches were conducted. A total of 263 studies were included in the second research. Our screening flow chart records the second search. The research included in this study is the result of the combination of the first retrieval and the second retrieval. The first search result is in the Supplementary data 2.

As depicted in Fig. 1, 154 studies were eliminated upon full-text screening. Finally, 263(combination of the results of two literature searches) articles that met our strict inclusion requirements were included in our analysis. This included 216 English articles and 47 Chinese articles. Moreover, 12 articles included two animal experiments [1, 36–45]. Among them, eight articles [39–46] included two investigations involving animal models of bone defects, one article [1] included two publications involving animal models of fracture disease and three articles [36–38] included one investigation of animal models of fracture and bone defect disease. At the end, we analyzed 275 animal experiments.

### The epidemiological characteristics of included studies

**Figure 2.**
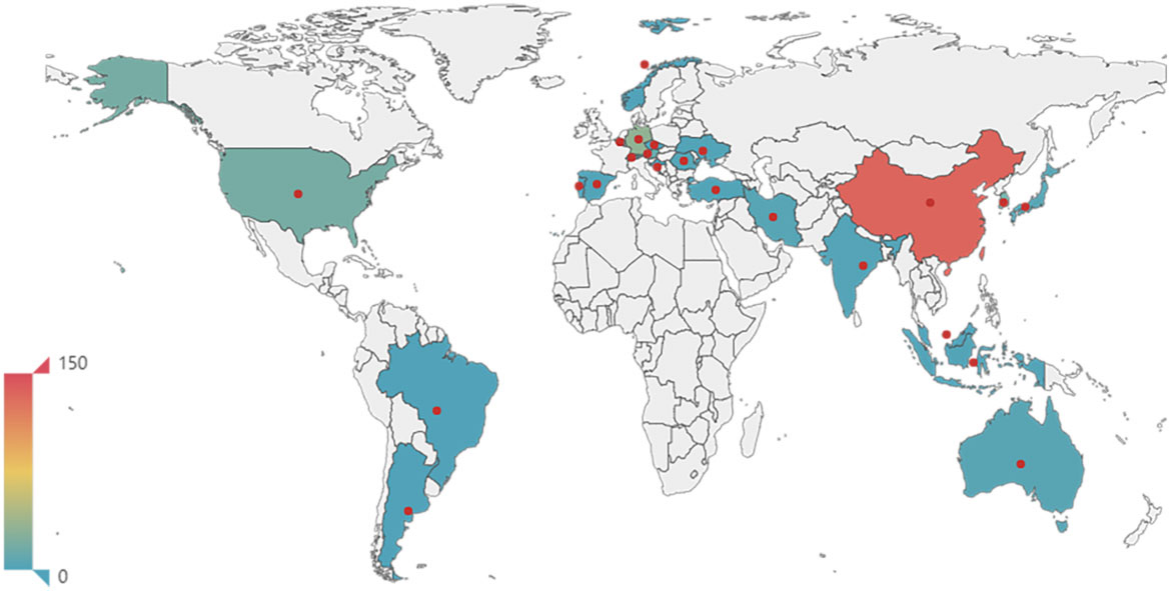
The country- and regional-based distribution of included studies.* *When calculating the percentage, the denominator was the number of documents included in this study, i.e. 263.

Among the 263 included studies, the earliest was published in 2006. In addition, China (134, 51.0%), Germany (32, 12.2%), South Korea (20, 7.6%), the USA (19, 7.2%) and Austria (13, 4.9%) published over 10 articles on our subject of interest (see Fig. 2), and the funding source was mostly non-profit (see Supplementary data 3).

### The major characteristics of included studies

In the included studies, rabbits (120, 43.6%) and rats (101, 36.7%) were the most commonly used species (Supplementary data 3). As depicted in Table 2, the average sample sizes of rabbits, rats and mice were 28, 33 and 23, respectively, and the median follow-up time (and IQR) were 42 (84–28), 42 (84–21) and 29 (84–15.8), respectively. The mean sample sizes of dogs, sheep and pigs were 12, 16 and 9, respectively, and the median follow-up time (and IQR) were 84 (112–56), 84 (168–42) and 84 (168–63), respectively.

**Table 2.** The animal species, sample size and duration of follow-up^a^

Animal species (*n*, %)		Sample size (x±S)^b^	Follow-up time [median (IQR)]^c^
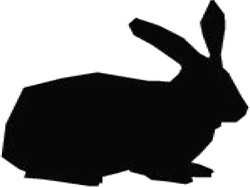 Rabbit	120 (43.6)	28 ± 21	42 (84–28)
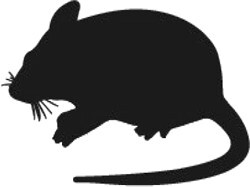 Rat	101 (36.7)	33 ± 27	42 (84–21)
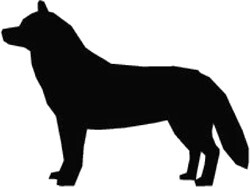 Dog	17 (6.2)	12 ± 7	84 (112–56)
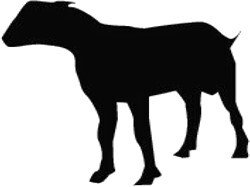 Sheep	15 (5.5)	16 ± 14	84 (168–42)
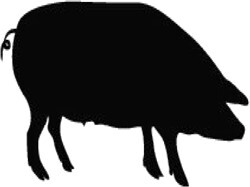 Swine	11 (4.0)	9 ± 5	84 (168–63)
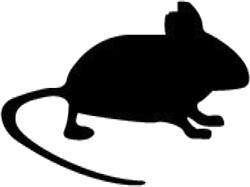 Mice	6 (2.2)	23 ± 15	29 (84–15.8)
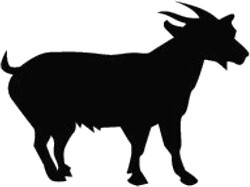 Goat^d^	3 (1.1)	–	56 (84–28)
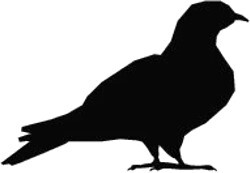 Bird^d^	1 (0.4)	–	–
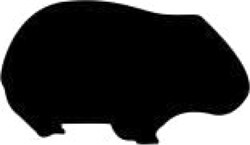 Guinea pig^d^	1 (0.4)	–	–

a When calculating the percentage, the denominator was the number of animal experiments included in this study, i.e. 275.

b The sample size of 12 experiments used rabbits, 11 experiments used rats, 2 experiments used dogs, 2 experiments used sheep and 2 experiments used mice were unclear or unreported.

c The follow-up time of rats used in three experiments and the follow-up time of rabbit in one experiment were unreported, and the unified unit of follow-up time is ‘day’.

d Since only three studies used goats, one study used birds and one experiment used guinea pigs, we did not conduct statistical analysis on the mean and standard deviation of the sample size of the two goat studies, and the follow-up time of birds was not included.

A total of 78.9% (217/275) of the included studies measured the outcome index of degradation, and 45.8% (126/275) measured gas formation (see Table 3). According to the published literature, most of the experiments using micro-CT and histological methods to measure implant degradation, while for the outcome index of gas formation, the most commonly used is general observation, followed by micro-CT and X-ray. In Supplementary data 4, we also provide more detailed information about the measurement of each outcome index, including the description method and presentation method of the outcome index. For example, for degradation, this index includes the presentation of specific indicators such as retention, degradation rate, corrosion rate and weight loss rate, etc.

**Table 3.** Measuring method and time point of degradation and gas formation outcome indicators^a^

Degradation (217, 78.9)		
Measuring method	(*n*, %)	Measurement time point [median (IQR)]
Micro-CT	125 (45.5)	56 (84–28)
Histological analysis	62 (22.5)	63 (112–28)
SEM and EDS	28 (10.2)	56 (84–28)
X-ray	27 (9.5)	28 (56–14)
Weight measurement	17 (6.2)	56 (84–28)
Others^b^	103 (37.5)	/

	Gas formulation (126, 45.8)	
Measuring method	(*n*, %)	Measurement time point [median (IQR)]^c^
General observation	41 (14.9)	/
Micro-CT	42 (15.7)	56 (84–21)
Histological analysis	27 (9.8)	56 (108.5–28)
X-ray	27 (9.8)	56 (112–28)
Radiography	17 (6.2)	52.5 (84–28)
Others^d^	19 (6.9)	/

a The denominator of all percentages in this table is the total number of experiments included in this study, i.e. 275.

b Due to the diversity of the measuring methods of degradation, many other measurement methods are included: CT, synchrotron radiation X-ray microscopy, high-resolution periodic quantitative computer tomography (HR pQCT), etc., we have not calculated the measurement time points of these measurement methods in a unified way. For details, please refer to our Supplementary data 4.

c Many experiments generally observed gas formation on every day of the study follow-up time, but on the other hand, many studies did not elaborate on the time point of observation. Therefore, we did not uniformly calculate the measurement time of this measurement method.

d In addition to the methods of measuring gas formation listed in Table 3, there are other methods used to measure this outcome index in the included studies, including: H2 sensors, syringe, MRI, etc., for the convenience of statistics, we classify this small number of measurement methods as ‘Others’, see the Supplementary data 4 for details.

Among the included studies, magnesium and magnesium-based materials were the most examined (242, 88.0%), followed by zinc and zinc-based materials (23, 8.4%), and iron and iron-based materials (10, 3.6%) (see Table 4). In terms of magnesium, the surface coating is a research hotspot. In our study, 34.5% (95/275) of eligible studies performed surface coating, including calcium phosphorus (17/97), micro-arc oxidation (14/97), plasma electrophoresis (8/97) and composite coatings (11/97) (Supplementary data 5).

**Table 4.** The types of degradable metal implant materials and control groups^a^

	**Types of degradable metal implant materials,** *n* (%)				
	Pure degradable metal	Alloy of degradable metal^b^	Surface coating of degradable metal and its alloy	Others^c^	Total
Mg	12 (4.4)	45 (16.4)	95 (34.5)	90 (32.7)	242 (88.0)
Zn	2 (0.7)	3 (1.1)	2 (0.7)	16 (5.8)	23 (8.4)
Fe	1 (0.4)	1 (0.4)	3 (1.1)	5 (1.8)	10 (3.6)
Total	15 (5.5)	49 (17.8)	100 (36.4)	111 (40.4)	233 (100)

Types of the control group (*n*, %)					
Positive control				Other types of control	
Degradable metals	Non-degradable metals	Degradable polymers	Other materials	Blank control^d^	Negative control
117 (42.4)	94 (34.1)	25 (9.1)	25 (9.1)	15 (5.4)	57 (20.7)

a The denominator of percentage calculation was 275.

b One study did not clearly indicate in the material section that the material used in the study was pure magnesium or magnesium alloy; one study did not specify in the materials section that the materials used in the study were pure iron or Fe alloy.

c ‘Others’ include composite materials and materials that physically modify the surface of degradable metal materials.

d Although the control group of five studies did not implant degradable metals into animals, based on the context, we could not determine whether fracture or bone defect or sham operation was performed; moreover, one study conducted the sham operation, and three studies did not report the material type of the control group.

As for the types of control groups (see Table 4), the degradable (117, 42.4%) and non-degradable metal materials (94, 34.1%) were the most commonly used positive control groups, and the negative control group (57, 20.7%) was the most commonly used among other types of control groups.

### Reporting quality assessment based on the ARRIVE guidelines 2.0

**Figure 3.**
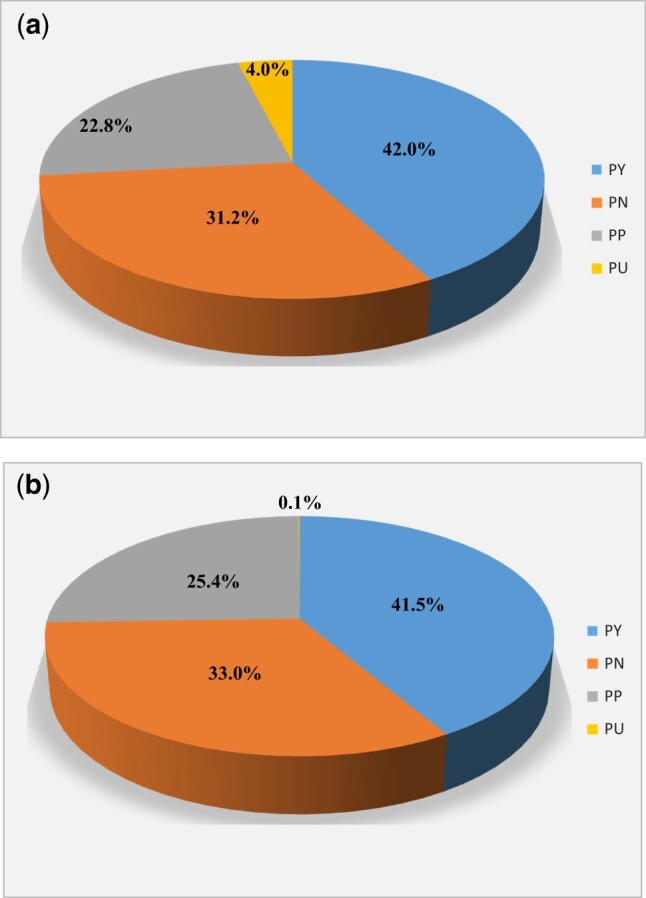
The overall reporting quality of the ARRIVE guidelines: (**a**) Essential 10. (**b**) Recommended set.

Generally, the reporting quality of preclinical animal experimentations in this field was relatively poor. As depicted in Fig. 3, in terms of the report quality evaluation of Essential 10 (22 sub-items), the overall coincidence rate was 42.0%. In addition, in terms of the ARRIVE Recommended Set (16 sub-items), the overall coincidence rate was 41.5%. Please refer to the Supplementary information for more details (Supplementary data 8–10).

#### The reporting quality of ARRIVE essential 10

**Figure 4.**
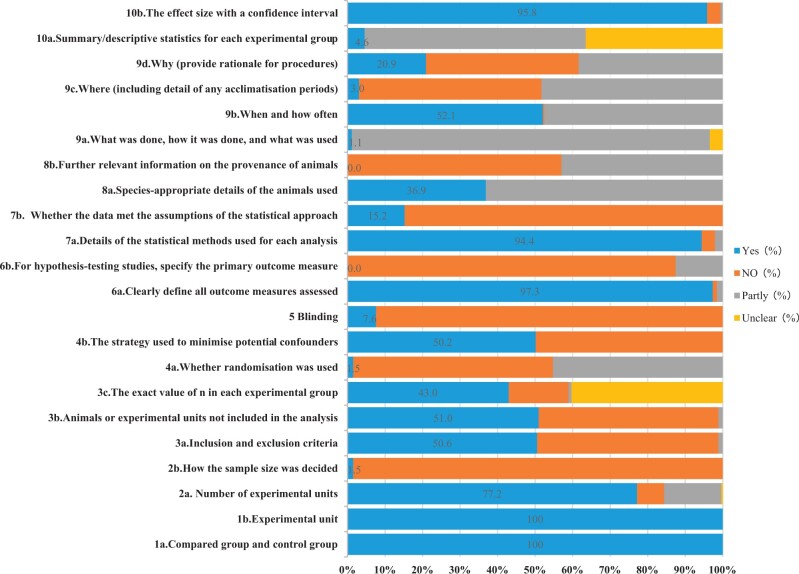
The quality of reporting and adherence to the ARRIVE essential 10 guidelines for the preclinical investigation of degradable metal research in animal models of bone defect and fracture.* *Since items 6b, 7 and 10b were not applicable to all included studies, the inapplicable studies were eliminated from the statistical analysis. Overall, 8 studies in item 6b, 197 studies in item 7, 192 studies in 10b participated in the final evaluation.

The study design, sample size, and inclusion/exclusion criteria (see Fig. 4): all included studies (263, 100%) provided brief details on the study design. Over half of these studies (203, 77.2%) specified the exact number of experimental units allocated to each group, and the total number in each experiment. However, most studies (259, 98.5%) did not explain how sample size was decided, and nearly half of the studies (126, 47.9%) failed to provide an exclusion criteria after modeling.

Randomization and blinding (see Fig. 4): over half of the studies (140, 53.2%) failed to report randomization, and nearly half of the studies (119, 45.2%) reported randomization, but did not provide the method. Most studies (243, 92.4%) also failed to report blinding.

The outcome measures and statistical methods(see Fig. 4): a vast majority of studies (256, 97.3%) clearly defined all assessed outcome measures. Among the included studies, eight studies were hypothesis-testing studies, and seven (87.5%) failed to specify the primary outcome measures (i.e. the outcome measure used to determine the sample size). Overall, 197 studies employed statistical methods, most of which (186, 94.4%) provided detailed information on the statistical methods used in each analysis, and a small portion of studies (30, 15.2%) described any methods used to assess whether the data met the assumptions of the statistical method.

Experimental animals and procedures (see Fig. 4): most studies (166, 63.1%) described the detailed information of experimental animals (species, strains and sub strains, gender, age or development stage, weight, etc.) incompletely. Nearly half of studies (113, 43.0%) partially described the provenance of animals, health/immune status, genetic modification status, genotype and previous procedures, and more than half (150, 57.0%) of the studies did not include any report on the above information. In general, the reporting quality of the included research on the item of experimental procedures was rather disappointing. Among them, the reporting quality on the sub-item ‘when’ was more comprehensive and detailed than other sub-items. In terms of the sub-item ‘what’, a fraction of studies (9, 3.4%) quoted their experimental procedures from prior investigations, but the extent to which they were quoted was not specified. This includes usage of narcotic drugs, anti-infective drugs, medical devices and equipment, as well as the member of the surgical team, so they received an ‘Unclear’ evaluation.

Results (see Fig. 4): more than half (155, 58.9%) of the studies reported only some representative results, and some (96, 36.5%) were judged as ‘Unclear’ because they failed to provide an inclusion/exclusion criteria, and the number of experimental animals per group in the final analysis.

#### The reporting quality of the ARRIVE recommended set

**Figure 5.**
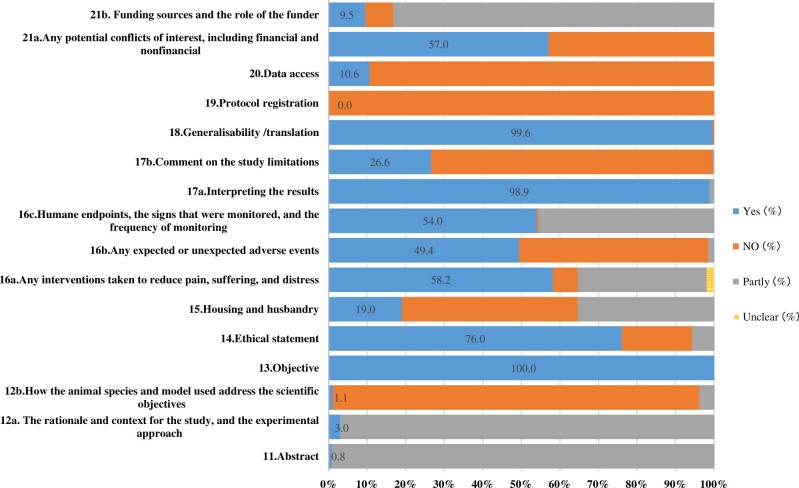
The quality of reporting and adherence to the ARRIVE recommended set for the preclinical evaluation of degradable metal research in animal models of bone defect and fracture.

The abstract, background and objectives (see Fig. 5): only a small number of studies (2, 0.8%) provided an accurate summary of the research objectives, animal species, strain and sex, key methods, main results and research conclusions. A vast majority of studies (255, 97.0%) did not fully describe the experimental animals or key methods. Most studies (250, 95.1%) failed to explain the experimental methods. In addition, only a few studies (3, 1.1%) described how the animal species and models were used to address the scientific objectives and, where appropriate, relevance to human biology. All included studies clearly described the research question, research objectives and specific research assumptions under investigation.

Housing, husbandry, animal care and monitoring (see Fig. 5): some studies (50, 19.0%) provided detailed information on housing and husbandry, such as cage/housings system, lighting, temperature and humidity, and so on, 35.4% (93/263) studies briefly described the above information, and nearly half of the studies (120, 45.6%) did not describe it at all. In addition, more than half of the studies (153, 58.2%) reported in detail the animal care and monitoring measures, but few studies (88, 33.5%) were still inadequate.

Interpretation/scientific implications and generalizability/translation (see Fig. 5): a vast majority of studies (260, 98.9%) took into account the research objectives and hypotheses, current theories and other relevant studies reported in the literature while interpreting the results. Most studies (192, 73.0%) did not include the limitations of the study, including potential sources of bias, limitations of animal models and inaccuracies related to the results. A vast majority of studies (262, 99.6%) reported whether and how the research results were transformed into other species or experimental conditions.

Ethical statement, declaration of interests, protocol registration, and data access (see Fig. 5): most studies (200, 76.0%) provided the name of the ethical review committee or equivalent institution that approved the use of animals in the study, as well as any relevant license or agreement number. Some studies (15, 5.7%) partially reported the information above (e.g. name of institution was missing), and a few studies (48, 18.3%) failed to involve any information involving ethical statement. Several studies (25, 9.5%) reported all funding sources (including grant identifiers) and the role of funders in research design, analysis and reporting. Most studies (219, 83.3%) only listed funding sources without description of the role of funds in research. None of the studies reported the status of the study registration plan. Finally, most studies (235, 89.4%) did not indicate whether and where the research data are available.

## Discussion

### Reporting quality is an important aspect that affects the translation of preclinical animal experiment

Compared with traditional materials, degradable metal materials have significant advantages in the field of fracture or bone defect repair [47]. Considering the advantages and disadvantages of various materials (degradation rate [48], mechanical properties [49] and biocompatibility [50]), researchers have carried out a lot of transformation and exploration work on degradable metal materials, mainly including alloying of the degradable metals [51], coating or surface modification [52] and the production of composite materials [53]. It is clear that the research results are remarkable [54, 55]. However, even though a large number of studies are in full swing, our previous work shows that the clinical translation, the inconsistency of conclusions and the quality of these studies are worrying [25, 33]. There are many reasons for concern. One is the quality of research methods. Degradation is an important aspect of evaluating the *in vivo* performance of implants in repairing fractures or bone defects [25, 33]. There are various methods to measure this outcome indicator. From the perspective of research design methods, different studies using different measurement methods at different time points will undoubtedly have an impact on the research results. For example, when evaluating degradation, descriptive general observation [52] and quantitative Micro-CT (calculation of degradation rate [56] (or corrosion rate [57]) and residual implant [58]) will certainly be different when drawing research conclusions. Another extremely important aspect is the quality of the reporting. Take degradation as an example. When measuring degradation, Micro-CT (125, 45.5%) is the most widely used method. However, the reason why this measuring method is used, and if this method is used, the reporting of residual implant [59], degradation rate [59] (or the corrosion rate [20]) is missing or incomplete will affect the reproducibility of following research on the one hand, and slacken the reliability of research results on the other hand. Ultimately, it will affect the translation of preclinical experiments.

### Poor reporting quality affects the evaluation of the internal validity of findings

The internal validity refers to the scientific robustness of research design, implementation, analysis and reporting [60]. According to our research, the reporting quality of the published animal research on the repair of bone defects/fractures using degradable metal materials was low. This may lead to the controversial evaluation of the reliability of research results, meaning, the internal validity was low. This enhances the risk of translating animal research results into clinical practice [10]. The sample size, randomization, blinding, statistical methods, experimental procedures, housing and husbandry of animal research are important factors that affect the internal validity of an animal experiment.

The sample size is crucial for the evaluation of statistical model validity and the robustness of the experimental results [10]. Too-small sample sizes may produce inconclusive results, and too large sample sizes may cause waste of resources and ethical concerns. Most included studies (203, 77.2%) reported the sample size. In general, the sample size of small animals was large (the average sample size of rabbits, rats and mice were 28, 33 and 23, respectively), and the sample size of large animals was relatively small (the average sample size of dogs, sheep and pigs were 12, 16 and 9, respectively). The discrepancy in sample sizes may be attributed to considerations, such as, cost, ethics and research purposes. However, only a few studies (4, 1.5%) explained the sample size determination method and its rationality. Interestingly, other known studies involving animal studies in hepatobiliary surgery [61], peritoneal dialysis [4] and rheumatology [3] also reached similar conclusions. The lack of reporting of the sample size determination method is a common problem in animal experimentations. The description of the sample size determination method is often based on the calculation of effect size and significance level [62]. If the former is not applicable, this can also be justified according to the research objectives [10]. To enhance the precision and reproducibility of an experiment as well as the reliability of the experimental results, it is necessary to conduct both scientific and rigorous reasoning and demonstration of the feasibility of the sample size. Subsequently, a comprehensive and carefully reporting of the sample size determination is absolutely crucial.

Randomization and blinding are significant strategies that reduce bias in research design. Using randomization during group allocation ensures that each experimental unit has an equal probability of receiving a particular treatment [10]. This helps minimize selection bias and reduce systematic differences in the characteristics of animals allocated to different groups. Blinding is helpful in reducing the subjective bias in the process of intervention implementation and result measurement. Among the included studies, the reports of randomization and blinding are not encouraging. Nearly half of the studies (119, 45.2%) reported randomization, but the randomization method was either unknown or lacked sufficient details, meaning that the conclusion may exaggerate the size of the effect [63]. Similarly, a study on the reporting quality of animal models involving cardiac arrest reached the same conclusions [64]. In terms of blinding, using the animal study of bone defects with degradable metal materials as an example [25], the measurement of new bone formation and bone defect healing mainly depend on the researchers’ observation of new bone formation around the implant, and bone defect healing via imaging and histological methods. If the observers were aware of the interventions received by the experimental animals prior to the analysis and/or interpretation of the results, they may have subjective measurement bias in evaluating the effect of osteogenesis or defect healing between groups, which may affect the validity of the data. Therefore, to obtain objective, accurate and reliable results, particularly those that depend on people’s subjective judgment, the effective implementation of blinding is absolutely essential to avoid subjective measurement bias.

In addition, most studies (186, 94.4%) provided detailed information on the statistical methods used in each analysis. However, a large number of studies (167, 84.8%) failed to describe the methods employed to evaluate whether the data met the assumptions of the statistical approach. The lack of test of normality and/or homogeneity of variance of data distribution was pretty common [20, 65]. A similar conclusion was reached in a quality reporting study involving peritoneal dialysis [4]. Hypothesis-testing is based on data assumptions. If the manuscript fails to describe how the assumptions are evaluated, and whether the data met these assumptions, readers or peers cannot fully evaluate the applicability of the employed statistical methods, which, ultimately, can affect reliability of results.

The experimental procedure is also a very crucial item in the guidelines. In the included studies, a detailed report on the experimental steps was missing. Taking the sub-item ‘9D. Why’, which explains the experimental steps as an example, only a few studies (55, 20.9%) explained specific procedures or techniques. There may be multiple approaches to investigating and evaluating a given research problem, so it is essential to explain why a specific program or technology was selected [10]. For example, for the measurement of results, some scholars [66] explained the reason why the displacement of the two patellar fragments was estimated by measuring the total length of the patella at different time points to identify the fracture space, instead of using radiological images. This not only provided a reference for later research, but also garnered support for the formation of subsequent evidence-based evidence.

### Poor reporting quality affects the external validity of a study

The external validity refers to the extent in which findings from one environment, population or species can be reliably applied to other environments, populations and species [67]. Currently, known factors threatening the external validity include experimental animal characteristics (age, sex, health status, microbiota and so on), as well as housing and husbandry [67]. Certainly, the external validity cannot be entirely improved due to species differences. However, it still possesses potentially modifiable characteristics like the representativeness of animal samples, animal husbandry, animal model characteristics and their clinical relevance [60].

In terms of the animal model characteristics and clinical relevance, researchers must select animals in relation to research objectives, compare the selected animals with human biological characteristics, fully describe the characteristics of experimental animals and extensively clarify the reasons behind the animal selection in the ‘Background’ section. Reporting animal characteristics is equivalent to the standardized human patient demographic data, which supports both the internal and external validity of the research [10]. Our research revealed that, at present, the reporting quality of clinical relevance of animal models in this field is not ideal, and less than half of the studies (97, 36.9%) reported the species, strain and sub-strain, gender, age or development stage, weight and so on of experimental animals in detail. However, further animal information (including animal origin, health/immune status, genetic modification status, genotype and previous procedures) were not addressed fully in any of the studies. Only a small portion of studies (3, 1.1%) described the reasons behind the selection of experimental animals.

Animal models can be divided into small (mice, rats, rabbits and so on) and large animal models (dogs, goats, pigs, sheep and so on). For ethical, economic and statistical reasons, small animal models are generally used during the preliminary evaluation of *in vivo* biomaterials [25, 33]. However, the clinical translation function of small animal models has great limitations. Therefore, typically, when a new bone graft material is introduced in clinic, the material must have undergone translation into large animal models. In this study, only a few studies employed large animals like dogs (17, 6.2%), sheep (15, 5.5%), pigs (11, 4.0%) and goats (3, 1.1%) to evaluate the properties of degradable metal materials. This may result in differences in the repair effects of degradable metals in preclinical animal experimentation and clinical trials. In addition to the animal species, the sex and age of animals also have a giant impact on the external validity of the experiment. Therefore, researchers must consider factors affecting the experimental result thoroughly, report the characteristics of experimental animals comprehensively and extensively explain the reasoning behind the selection of animals in the ‘Background’ section.

Animal housing and husbandry are another important element that affects external validity. Taking the feeding of rats as an example [68], one of the most common incomplete information in our selected articles was the description of animal feeding, such as, ‘standard rat chow and water were provided *ad libitum*’. This inaccurate description may endanger the external validity because different feeding may affect the results.

### Poor reporting quality affects the reproducibility of animal experimentation

‘Reproducibility’ encompasses three aspects: reanalysis of existing datasets (‘the reproducibility of analysis’); collection of a similar quantity of new data as the first experiment (‘the reproducibility of experimental results’); and, the deliberate alteration of the experimental conditions or analytical methods to determine the same conclusion as before (‘robustness’) [69]. Preclinical research, particularly, research using animal models, is considered to be the area most vulnerable to reproducibility challenges [9]. A series of complex factors result in the lack of reproducibility, among which is the lack of transparent and accurate reporting.

A study comparing the therapeutic effects of peri-implant infection in animals and humans found that the therapeutic effects reported in animal studies were far greater than those reported in human studies. Moreover, there was a lack of homogeneity in research design and data analyses between animal studies and human studies [70]. It is likely that missing reports on important information like measurement methods, results and so on may be one of the reasons behind the discrepancy between animal and human study conclusions. This is corroborated in other disciplines, namely, rheumatology [3], peritoneal dialysis [4] and otorhinolaryngology [7]. In the field of animal experiments involving fracture/bone defects repair using degradable metal materials, the low reproducibility of animal experiments and inconsistent conclusions in existing studies is of major concern [25, 33]. Our research also revealed that the reporting quality of animal experiments in this field was unsatisfactory. The reporting of important information was opaque and incomplete, which can introduce potential bias when peers conduct research based on prior investigations. Eventually, this can lead to the low quality of follow-up research reports, reduced reliability of research results, inconsistency between research results and previous studies, and reduced reproducibility. For example, in the process of evaluation, we observed that the experimental procedures of certain studies [71] were quoted from previous publications, while the basic elements of the experimental design of the referenced research itself had incomplete and opaque information, so the reliability of the obtained experimental results and conclusions from this type of research is somewhat controversial.

In short, the reporting of each item typically affects translation from animal experiments to clinical practice, from one or multiple levels at the same time. In face of insufficient and opaque information reporting, readers are likely not sure whether the reason for the incomplete information is sufficient method but insufficient reporting or whether it is insufficient method [64]. The research design, implementation and analysis of scientific research are presented in the step ‘report’. Therefore, it is necessary to conduct a scientific, stable and transparent reporting to improve the internal/external validity and reproducibility, and thereby, enhance the translation of preclinical research.

Hence, we believe that, when it comes to the evaluation of the reporting quality of studies, although ARRIVE guidelines 2.0 provide a ‘common’ standardized guidance for the report of preclinical animal experiments, there is still a lack of ‘personality’ of the report of preclinical animal experiments involving degradable metal materials for the repair of fractures/bone defects. This can potentially lead to certain restrictions on the application of this guideline in the field. For example, a surface coating of degradable metal materials can markedly improve corrosion resistance [72], biocompatibility [73] and ability to stimulate new bone formation [74]. However, some crucial elements regarding the coating, such as, corrosion rate, surface chemistry, adhesion, coating morphology and controllability of degradation, were not fully discussed in numerous studies [75]. In addition, the research design involving animal experimentations in materials science must be guided by the objectives of the experiment, including, the selection of the experimental animals (such as, the selection of large animals [76] versus small animals [77]), the control group setting (such as, selection of materials in the positive control group), the determination of follow-up time (in our analyzed studies, the experimental cycle was at least 0 days [78], and the longest was 504 days [79]), the experimental results (the effectiveness [31] or safety index [80] or both [65]) and the measurement method of experimental results (e.g. the *in vivo* measurement of hydrogen production by magnesium includes naked eye observation [81], syringe suction measurement [82], imaging observation [83] and so on), which must be reported in detail, and the rationality and reliability must be demonstrated in the ‘Background’ or ‘Method’ section. Therefore, we suggest following the filling, improvement, expansion and extension of the ARRIVE guidelines 2.0 in the field of materials science in the future. To ensure the overall improvement of quality reporting, we also believe that we must not only focus on the complete reporting of materials and animal experimentations, but also make great efforts toward the reporting of specific diseases. For example, in terms of the preclinical animal experiment involving bone defects repair with degradable metal materials, researchers must not only ensure the transparent, accurate and comprehensive reporting of degradable metal materials and animal experiments, but also ensure the complete reporting of the bone defect itself, such as, defect site, defect size, osteotomy method and so on [84]. Moreover, this needs to be the criteria for reporting in other fields of study as well.

Being a guiding document that instructs researchers and publishers to clearly and accurately report and publish the design, implementation process and all results of medical research, the ARRIVE guidelines not only provides a basic reporting standard, but also puts forward constructive suggestions for improving the overall quality of preclinical animal experimentation reports, such as, data access, protocol registration and so on. Our research revealed that the reporting quality of protocol registration, data access (the non-coincidence rate was 100% and 89.4% respectively) and other items were low in the selected studies. The data access and protocol registration platforms are readily available [85]; however, their application is not extensive. We, therefore, suggest that all parties strictly implement the ARRIVE guidelines, and improve measures that enhances the transparency of reporting, including, data access, protocol registration and so on.

### Limitations of this study

This study has several limitations. First, the purpose of this study was to assess the reporting quality of animal experiments in the field of material science. However, we did not evaluate the methodological or overall quality of our selected studies [3, 5, 6]. Second, the ARRIVE guidelines evaluation process is subjective. Hence, even if we conducted a pilot experiment prior to commencing our independent assessment of the reporting process, the subjective bias may not be completely avoided. Third, we only included articles in Chinese or English, so the conclusion may not necessarily be applicable to the research of other languages.

## Conclusion

Our analyses suggested that the reporting quality of the published animal experiments involving fracture/bone defects repair using degradable metal materials is rather disappointing. There was a significant lack of transparent, accurate and comprehensive reporting on key elements of experimental design (such as, sample size and its calculation, randomization, blinding, statistical method and so on), as well as other elements that avoid bias (such as, declaration of interest, funding source, data access, protocol registration and so on). Given our conclusion, we suggest that it is important to further promote the use of the ARRIVE guidelines 2.0 in professional journals and advocate researchers to strictly abide by it, appropriately supplement the contents of the ARRIVE guidelines items in the manuscript review and instructions for authors [3, 7, 86], and draw lessons from the worldwide promotion of CONSORT statement: i.e., the ARRIVE guidelines can be promoted through expert lectures and articles and introductions in high impact journals, etc. [7, 12, 18]. Meanwhile, it is necessary to encourage the provision of raw online data to support papers [9], and launch the registration program of preclinical animal experiments, to improve the transparency and reproducibility of biological science research, and promote the welfare of research animals.

## Supplementary data

Supplementary data are available at *REGBIO* online.

## Funding

This work was supported by the National Natural Science Foundation of China [No. 81873184] and National Key R&D Program of China [No. 2018YFC1106700].

*Conflicts of interest statement*. None declared.

## Supplementary Material

rbac076_Supplementary_Data
