# Supplementary material for: Quality of reporting and adherence to the ARRIVE guidelines 2.0 for preclinical degradable metal research in animal models of bone defect and fracture: a systematic review
Source: Regen Biomater. 2022 Oct 3;9:rbac076. doi: 10.1093/rb/rbac076 (PMC9632456; doi:10.1093/rb/rbac076)
Supplement: rbac076_Supplementary_Data [file rbac076_supplementary_data.zip › Supplementary data 7.docx]

Types of control group：

Positive control: ① degradable metal materials, such as magnesium and magnesium based materials; ② Non-degradable metal materials, such as titanium and stainless steel; ③ Degradable polymers, such as polylactic acid; ④ Others, such as autologous bone, composite materials, etc；

Other types of control: ① blank control (no drilling, no implantation); ② Negative control (drilled, not implanted); ③ Sham operation (no fracture or bone defect, but other operations were performed).
