# Supplementary material for: Quality of reporting and adherence to the ARRIVE guidelines 2.0 for preclinical degradable metal research in animal models of bone defect and fracture: a systematic review
Source: Regen Biomater. 2022 Oct 3;9:rbac076. doi: 10.1093/rb/rbac076 (PMC9632456; doi:10.1093/rb/rbac076)
Supplement: rbac076_Supplementary_Data [file rbac076_supplementary_data.zip › Supplementary data 1.docx]

**First retrieval：**

**Pubmed：20211007**

1. (biodegradable metal OR degradable metal OR biodegradable alloy OR degradable alloy OR absorbable metal) OR ((biodegradable implants OR biodegradable fixation OR absorbable implants OR bioabsorbable implants OR biodegrading implants) AND (metal OR alloy OR magnesium OR Mg OR zinc OR Zn OR Iron OR Fe)) [tiab] (26,319)
2. "Fractures, Bone"[Mesh] OR fracture*[tiab] (322,902)
3. Bone defect*[tiab] (15,165)
4. "Fracture Healing"[Mesh] OR "Fracture Healing"[tiab] OR "fracture fixation"[Mesh] OR ("fracture"[tiab] AND "fixation"[tiab]) OR "fracture fixation"[tiab] (88,914 )
5. Bone repair*[tiab] OR bone heal[tiab] OR bone healed[tiab] OR bone heals[tiab] OR bone healing[tiab] (24,591)
6. Bone fill*[tiab] (1,241 )
7. "Bone Screws"[Mesh] OR "Bone Screws"[tiab] OR "Bone Plates"[Mesh] OR "Bone Plates"[tiab] OR "Bone Nails"[Mesh] OR "Bone Nails"[tiab] OR intramedullary nail*[tiab] OR "pins"[tiab] (57,350)
8. #2 OR #3 OR #4 OR #5 OR #6 OR #7 (375,093)
9. Search filter for animal studies [1] (7,404,572)
10. #1 AND #8 AND #9 (544)

**Embase:**

1.(biodegradable metal or degradable metal or biodegradable adj3 alloy or degradable adj3 alloy or absorbable metal or ((biodegradable implants or biodegradable fixation or absorbable implants or bioabsorbable implants or biodegrading implants) and (metal or alloy or magnesium or Mg or zinc or Zn or Iron or Fe))).mp. (2457)

2.exp fracture/ (352，696)

3.exp bone defect/ (14，156)

4.exp fracture healing/ or exp fracture fixation/ (133，768)

5.exp bone filler/ (536)

6.exp bone screws/ or exp bone plates/ or exp bone nails/ (61，754)

7.(fractur$ or (bone adj3 defect$)).mp. (417，767)

8.(fracture healing or fracture fixation or (bone adj3 fill$) or bone screw$ or bone plate$ or bone nail$ or pins or (bone adj3 repair$) or (bone adj3 heal$)).mp. (27，407)

9.or/2-8 (485，074)

10. Search filter for animal studies [2] (29，808，991)

And/1,9,10 (719)

**Cochrane library：**

1. ((biodegradable metal OR degradable metal OR biodegradable near/3 alloy OR degradable alloy OR absorbable metal) OR ((biodegradable implants OR biodegradable fixation OR absorbable implants OR bioabsorbable implants OR biodegrading implants) AND (metal OR alloy OR magnesium OR Mg OR zinc OR Zn OR Iron OR Fe))):ti,ab,kw (569)
2. MeSH descriptor: [Fractures, Bone] explode all trees (6509)
3. MeSH descriptor: [Fracture Healing] explode all trees (549)
4. MeSH descriptor: [Fracture Fixation] explode all trees (1852)
5. (fracture* or (bone near/3 defect*) or (Fracture Healing) or (Fracture Fixation) or (bone near/3 repair*) or (bone near/3 fill*) or (bone near/3 heal*)):ti,ab,kw (27436)
6. MeSH descriptor: [Bone Screws] explode all trees (838)
7. MeSH descriptor: [Bone Plates] explode all trees (640)
8. MeSH descriptor: [Bone Nails] explode all trees (444)
9. (Bone Screw* or Bone Plate* or Bone Nail* or pins or intramedullary nail*):ti,ab,kw (9116)
10. #2 or #3 or #4 or #5 or #6 or #7 or #8 or #9 (33034)
11. (animal* or animal experiment or animal stud* or animal trail or nonhuman or rat* or mice or pig* or swine* or dog* or sheep* or goat* horse* or oranguta* or monkey) (624169)
12. #1 and #10 and #11 (40)

**Web of science**：

1.TS=(biodegradable metal OR degradable metal OR Biodegradable alloy OR degradable alloy OR absorbable metal) OR (TS=(biodegradable implants OR Biodegradable fixation OR absorbable implants OR bioabsorbable implants OR biodegrading implants) AND TS=(metal OR alloy OR magnesium OR magnesia OR zinc OR Zn OR Iron OR Fe)) (13767)

2.TS=(fracture* OR bone defect*) OR TS=(Fracture Healing or Fracture Fixation or bone repair* OR bone fill* OR bone heal OR bone healed OR bone heals OR bone healing) OR TS=(Bone Screw* or Bone Plate* or Bone Nail* or pins or intramedullary nail*) (1386116)

3.TS=(animal* or animal experiment or animal stud* or animal trail or nonhuman or rat* or mice or pig* or swine* or dog* or sheep* or goat* horse* or oranguta* or monkey) (29389809)

4.#1 AND #2 AND #3 (1582)

**CNKI**：

(SU % '可降解金属' OR SU % '可降解合金' OR SU % '镁合金' OR SU % '锌合金' OR SU % '铁合金' OR SU = '生物可降解固定物' OR SU % '可吸收植入物' OR SU = '可降解植入物') AND (SU = '骨折' OR SU = '骨缺损' OR SU = '骨折愈合' OR SU = '骨折固定' OR SU = '骨修复' OR SU = '骨填充' OR SU = '骨钉' OR SU = '骨板' OR SU = '骨针') AND (FT = '动物实验' OR FT = '动物' OR FT = '鼠' OR FT = '羊' OR FT = '猪' OR FT = '犬' OR FT = '牛' OR FT = '猩猩' OR FT = '马' OR FT = '猴') (230)

**WanFang:**

(主题: (生物可降解金属 OR 可降解金属 OR 生物可降解合金 OR 可降解合金 OR 可降解镁合金 OR 可降解锌合金 OR 可降解铁合金) OR 主题:(生物可降解植入物 OR 生物可降解固定物 OR 可吸收植入物 OR 生物可吸收植入物 OR 可降解植入物)) AND 主题: ("骨折" OR "骨缺损" OR "骨折愈合" OR "骨折固定" OR "骨修复" OR "骨填充" OR "骨钉" OR "骨板" OR "骨针") AND 主题: ("动物" OR "动物实验" OR "鼠" OR "羊" OR "猪" OR "犬" OR "牛" OR "猩猩" OR "马" OR "猴") (530)

**VIP:**

M=(生物可降解金属 OR 可降解金属 OR 生物可降解合金 OR 可降解合金 OR 可降解镁合金 OR 可降解锌合金 OR 可降解铁合金 OR 生物可降解植入物 OR 生物可降解固定物 OR 可吸收植入物 OR 生物可吸收植入物) AND M=(骨折 OR 骨缺损 OR 骨折愈合 OR 骨折固定 OR 骨修复 OR 骨填充 OR 骨钉 OR 骨板 OR 骨针) AND U=(动物 OR 动物实验 OR 鼠 OR 羊 OR 猪 OR 犬 OR 牛 OR 猩猩 OR 马 OR 猴) (8)

**CBM:**

( "生物可降解金属"[常用字段:智能] OR "可降解金属"[常用字段:智能] OR "生物可降解合金"[常用字段:智能] OR "可降解合金"[常用字段:智能] OR "镁合金"[常用字段:智能] OR "锌合金"[常用字段:智能] OR "铁合金"[常用字段:智能] OR "生物可降解植入物"[常用字段:智能] OR "生物可降解固定物"[常用字段:智能] OR "可吸收植入物"[常用字段:智能] OR "生物可吸收植入物"[常用字段:智能]) AND( "骨折"[常用字段:智能] OR "骨缺损"[常用字段:智能] OR "骨折愈合"[常用字段:智能] OR " 骨折固定"[常用字段:智能] OR "骨修复"[常用字段:智能] OR "骨填充OR 骨钉"[常用字段:智能] OR "骨板"[常用字段:智能] OR "骨针"[常用字段:智能]) AND( "动物"[常用字段:智能] OR "动物实验"[常用字段:智能] OR "鼠"[常用字段:智能] OR "羊"[常用字段:智能] OR "猪"[常用字段:智能] OR "犬OR 牛"[常用字段:智能] OR "猩猩"[常用字段:智能] OR "马"[常用字段:智能] OR "猴"[常用字段:智能]) (17)

**Second retrieval**

Pubmed：20220614

1. (biodegradable metal OR degradable metal OR biodegradable alloy OR degradable alloy OR absorbable metal) OR ((biodegradable implants OR biodegradable fixation OR absorbable implants OR bioabsorbable implants OR biodegrading implants) AND (metal OR alloy OR magnesium OR Mg OR zinc OR Zn OR Iron OR Fe)) (770,893)

2. "Fractures, Bone"[Mesh] OR fracture*[tiab] (334,736)

3. Bone defect*[tiab] (16,265)

4. "Fracture Healing"[Mesh] OR "Fracture Healing"[tiab] OR "fracture fixation"[Mesh] OR ("fracture"[tiab] AND "fixation"[tiab]) OR "fracture fixation"[tiab] (92,211)

5. Bone repair*[tiab] OR bone heal[tiab] OR bone healed[tiab] OR bone heals[tiab] OR bone healing[tiab] (25,761)

6. Bone fill*[tiab] (1,278)

7. "Bone Screws"[Mesh] OR "Bone Screws"[tiab] OR "Bone Plates"[Mesh] OR "Bone Plates"[tiab] OR "Bone Nails"[Mesh] OR "Bone Nails"[tiab] OR intramedullary nail*[tiab] OR "pins"[tiab] (59,561 )

8. "Bone Regeneration"[Mesh] OR "Bone Regeneration"[tiab] OR "Osteoconduction"[tiab] OR "bone tissue regeneration"[tiab] (36,299)

9. #2 OR #3 OR #4 OR #5 OR #6 OR #7 OR #8 (412,099)

10. Search filter for animal studies [1] (7,404,572)

11. #1 AND #9 AND #10 (3,121)

12. #1 AND #9 AND #10（1954~2021） (3,085)

Embase:

1.(biodegradable metal or degradable metal or biodegradable near/3 alloy or degradable near/3 alloy or absorbable metal or ((biodegradable implants or biodegradable fixation or absorbable implants or bioabsorbable implants or biodegrading implants) and (metal or alloy or magnesium or Mg or zinc or Zn or Iron or Fe))) : mapping (Map to preferred term in Emtree; Search also as free text in all fields; Explode using narrower Emtree terms; Search as broadly as possible) (8,822)

2.exp fracture/ (366,401)

3.exp bone defect/ (14,929)

4.exp fracture healing/ or exp fracture fixation/ (138,811)

5.exp bone filler/ (595)

6.exp bone screws/ or exp bone plates/ or exp bone nails/ (65,378)

7.(fractur* or (bone near/3 defect*)): mapping (Map to preferred term in Emtree; Search also as free text in all fields; Explode using narrower Emtree terms; Search as broadly as possible) (488,237)

8.(fracture healing or fracture fixation or (bone near/3 fill*) or bone screw* or bone plate* or bone nail* or pins or (bone near/3 repair*) or (bone near/3 heal*)): mapping (Map to preferred term in Emtree; Search also as free text in all fields; Explode using narrower Emtree terms; Search as broadly as possible) (82，931)

9. exp bone regeneration/ (34,798)

10. ((bone tissue regeneration) or (bone regeneration)): mapping (Map to preferred term in Emtree; Search also as free text in all fields; Explode using narrower Emtree terms; Search as broadly as possible) (113,234)

11.or/2-10 (653,117)

12. Search filter for animal studies [2] (30，850，073)

13. And/1,11,12 (1648) (Among them, the first one was published in 1973)

14. #1 AND #11 AND #12 AND [1973-2021]/py (1567) (1567)

Cochrane library：

1. ((biodegradable metal OR degradable metal OR biodegradable near/3 alloy OR degradable alloy OR absorbable metal) OR ((biodegradable implants OR biodegradable fixation OR absorbable implants OR bioabsorbable implants OR biodegrading implants) AND (metal OR alloy OR magnesium OR Mg OR zinc OR Zn OR Iron OR Fe))):ti,ab,kw (572)

2. MeSH descriptor: [Fractures, Bone] explode all trees (6790)

3. MeSH descriptor: [Fracture Healing] explode all trees (558)

4. MeSH descriptor: [Fracture Fixation] explode all trees (1929)

5. (fracture* or (bone near/3 defect*) or (Fracture Healing) or (Fracture Fixation) or (bone near/3 repair*) or (bone near/3 fill*) or (bone near/3 heal*)):ti,ab,kw (28455)

6. MeSH descriptor: [Bone Screws] explode all trees (869)

7. MeSH descriptor: [Bone Plates] explode all trees (669)

8. MeSH descriptor: [Bone Nails] explode all trees (451)

9. (Bone Screw* or Bone Plate* or Bone Nail* or pins or intramedullary nail*):ti,ab,kw (9562)

10. MeSH descriptor: [bone regeneration] explode all trees (891)

11. (bone regeneration* or bone tissue regeneration*):ti,ab,kw (2013)

12. #2 or #3 or #4 or #5 or #6 or #7 or #8 or #9 or #10 or #11 (35657)

13. (animal* or animal experiment or animal stud* or animal trail or nonhuman or rat* or mice or pig* or swine* or dog* or sheep* or goat* horse* or oranguta* or monkey) (645766)

14. #1 and #12 and #13 (1994~2021) (44)

Web of science：

1.TS=(biodegradable metal OR degradable metal OR Biodegradable alloy OR degradable alloy OR absorbable metal) OR (TS=(biodegradable implants OR Biodegradable fixation OR absorbable implants OR bioabsorbable implants OR biodegrading implants) AND TS=(metal OR alloy OR magnesium OR magnesia OR zinc OR Zn OR Iron OR Fe)) (24,303)

2.TS=(fracture* OR bone defect*) OR TS=(Fracture Healing or Fracture Fixation or bone repair* OR bone fill* OR bone heal OR bone healed OR bone heals OR bone healing OR bone regeneration OR bone tissue regeneration) OR TS=(Bone Screw* or Bone Plate* or Bone Nail* or pins or intramedullary nail*) (3,271,610)

3.TS=(animal* or animal experiment or animal stud* or animal trail or nonhuman or rat* or mice or pig* or swine* or dog* or sheep* or goat* horse* or oranguta* or monkey) (37,276,499)

4.#1 AND #2 AND #3 (2,268) Among them, the first one was published in 1973

5. #1 AND #2 AND #3 (1945-2021) ([2,107](https://www.webofscience.com/wos/alldb/summary/92d62539-e88c-4c79-8dda-517a704fa478-3de54714/date-ascending/1))

CNKI：

(SU % '可降解金属' OR SU % '可降解合金' OR SU % '镁合金' OR SU % '锌合金' OR SU % '铁合金' OR SU = '生物可降解固定物' OR SU % '可吸收植入物' OR SU = '可降解植入物') AND (SU = '骨折' OR SU = '骨缺损' OR SU = '骨折愈合' OR SU = '骨折固定' OR SU = '骨修复' OR SU = '骨填充' OR SU = '骨钉' OR SU = '骨板' OR SU = '骨针' OR SU = '骨再生' OR SU = '骨组织再生') AND (FT = '动物实验' OR FT = '动物' OR FT = '鼠' OR FT = '羊' OR FT = '猪' OR FT = '犬' OR FT = '牛' OR FT = '猩猩' OR FT = '马' OR FT = '猴') (263) （~2021）

WanFang:

(主题: (生物可降解金属 OR 可降解金属 OR 生物可降解合金 OR 可降解合金 OR 可降解镁合金 OR 可降解锌合金 OR 可降解铁合金) OR 主题:(生物可降解植入物 OR 生物可降解固定物 OR 可吸收植入物 OR 生物可吸收植入物 OR 可降解植入物)) AND 主题: ("骨折" OR "骨缺损" OR "骨折愈合" OR "骨折固定" OR "骨修复" OR "骨再生" OR "骨组织再生" OR "骨钉" OR "骨板" OR "骨针" OR "骨填充") AND 主题: ("动物" OR "动物实验" OR "鼠" OR "羊" OR "猪" OR "犬" OR "牛" OR "猩猩" OR "马" OR "猴") (~2021) (634)

VIP:

M=(生物可降解金属 OR 可降解金属 OR 生物可降解合金 OR 可降解合金 OR 可降解镁合金 OR 可降解锌合金 OR 可降解铁合金 OR 生物可降解植入物 OR 生物可降解固定物 OR 可吸收植入物 OR 生物可吸收植入物) AND M=(骨折 OR 骨缺损 OR 骨折愈合 OR 骨折固定 OR 骨修复 OR 骨再生 OR 骨组织再生 OR 骨填充 OR 骨钉 OR 骨板 OR 骨针) AND U=(动物 OR 动物实验 OR 鼠 OR 羊 OR 猪 OR 犬 OR 牛 OR 猩猩 OR 马 OR 猴) (6)

CBM:

( "生物可降解金属"[常用字段:智能] OR "可降解金属"[常用字段:智能] OR "生物可降解合金"[常用字段:智能] OR "可降解合金"[常用字段:智能] OR "镁合金"[常用字段:智能] OR "锌合金"[常用字段:智能] OR "铁合金"[常用字段:智能] OR "生物可降解植入物"[常用字段:智能] OR "生物可降解固定物"[常用字段:智能] OR "可吸收植入物"[常用字段:智能] OR "生物可吸收植入物"[常用字段:智能]) AND( "骨折"[常用字段:智能] OR "骨缺损"[常用字段:智能] OR "骨折愈合"[常用字段:智能] OR " 骨折固定"[常用字段:智能] OR "骨修复"[常用字段:智能] OR "骨填充OR 骨钉"[常用字段:智能] OR "骨板"[常用字段:智能] OR "骨针"[常用字段:智能] OR "骨再生"[常用字段:智能] OR "骨组织再生"[常用字段:智能]) AND( "动物"[常用字段:智能] OR "动物实验"[常用字段:智能] OR "鼠"[常用字段:智能] OR "羊"[常用字段:智能] OR "猪"[常用字段:智能] OR "犬OR 牛"[常用字段:智能] OR "猩猩"[常用字段:智能] OR "马"[常用字段:智能] OR "猴"[常用字段:智能]) (17)
